# The Physiological Effects of Whole-Body Vibration Combined with Other Exercise Modalities in Overweight and Obese Individuals: A Systematic Review

**DOI:** 10.3390/biology14060711

**Published:** 2025-06-17

**Authors:** Daniel Batouli-Santos, Ana Carolina Coelho-Oliveira, Vanessa Amaral Mendonça, Alexei Wong, Adérito Seixas, Ana Cristina Rodrigues Lacerda, Anelise Sonza, Ayman Alhammad, Mario Bernardo-Filho, Danúbia da Cunha de Sá-Caputo, Redha Taiar

**Affiliations:** 1 Laboratório de Vibrações Mecânicas e Práticas Integrativas, Departamento de Biofísica e Biometria, Instituto de Biologia Roberto Alcantara Gomes, Policlínica Universitária Piquet Carneiro, Universidade do Estado do Rio de Janeiro, Rio de Janeiro 20551-030, RJ, Brazil; danielbatouli@gmail.com (D.B.-S.); anacarol_coelho@hotmail.com (A.C.C.-O.); bernardofilhom@gmail.com (M.B.-F.); dradanubia@gmail.com (D.d.C.d.S.-C.); 2 Programa de Pós-Graduação em Fisiopatologia Clínica e Experimental, Faculdade de Ciências Médicas, Universidade do Estado do Rio de Janeiro, Rio de Janeiro 20551-030, RJ, Brazil; 3 Faculdade de Ciências Biológicas e da Saúde, Universidade Federal dos Vales do Jequitinhonha e Mucuri (UFVJM), Diamantina 39100-000, MG, Brazil; vaafisio@hotmail.com (V.A.M.); lacerdaacr@gmail.com (A.C.R.L.); 4 Department of Health and Human Performance, Marymount University, Arlington, VA 22207, USA; awong@marymount.edu; 5 FP-I3ID, FP-BHS, Escola Superior de Saúde Fernando Pessoa, 4200-256 Porto, Portugal; aderito@ufp.edu.pt; 6 LABIOMEP, INEGI-LAETA, Faculdade de Desporto, Universidade do Porto, 4099-002 Porto, Portugal; 7 Departamento de Fisioterapia, Programa de Pós-Graduação em Fisioterapia and Programa de Pós-Graduação em Ciências do Movimento Humano, Universidade do Estado de Santa Catarina—UDESC, Florianópolis 88035-901, SC, Brazil; anelise.sonza@udesc.br; 8 Department of Physiotherapy, College of Medical Rehabilitation Sciences, Taibah University, Al-Madinah Al-Munawarrah 41477, Saudi Arabia; aalhamad@taibahu.edu.sa; 9 Matériaux et Ingénierie Mécanique, Université de Reims Champagne Ardenne, 51687 Reims, France

**Keywords:** whole-body vibration, systemic vibratory therapy, exercise therapy, overweight, obesity

## Abstract

Introduction: Emerging evidence indicates that whole-body vibration (WBV) may be a relevant adjunct in interventions targeting overweight and obese individuals. Moreover, WBV has been applied in combination with other exercise modalities and therapeutic strategies, with various physiological responses observed in this population. Objective: The current systematic review aimed to assess the physiological effects of WBV with other modalities of exercises in overweight and obese individuals. Methods: Searches in PubMed, Embase, Web of Science, Scopus, and Cochrane databases were conducted to assess physiological responses to the combination of WBV with other modalities of exercises in overweight and obese individuals. Publications were identified and the screening was performed by two reviewers, independently. Irrelevant studies were excluded based on the eligibility criteria. Results: Seven studies, analyzing different outcomes, were included, with a mean methodological quality score of 7. Four studies analyzed the acute effect and three studied the cumulative effect, with different intervention protocols. Increases in growth hormone (GH) concentrations and bioelectrical phase angles, and reductions in fat mass, blood triglycerides, and cholesterol concentrations, as well as blood pressure, heart rate, and arterial stiffness, were reported. Conclusions: WBV combined with other exercise modalities seems to promote important physiological responses in overweight and obese individuals. However, more large-scale, long-term randomized controlled trials with WBV and other modalities of exercises in overweight and obese cohorts are needed to corroborate and expand the findings of the current systematic review.

## Simple Summary

Obesity is a risk factor for the development of several diseases. Physical exercise is one of the main forms of prevention and treatment. However, obese individuals may have difficulty or lack motivation to adhere to a regular physical exercise program. Therefore, it is important to offer different types of exercises with the aim of increasing the level of daily physical activity of these individuals. Whole-body vibration is an alternative, with low perceived exertion and with several benefits reported in the literature, such as reductions in body fat mass and the concentration of triglycerides and blood cholesterol. Whole-body vibration combined with other types of exercise may be another option to collaborate in the treatment of obesity. The objective of this review was to summarize the effects of whole-body vibration combined with other types of exercise in overweight and obese individuals, so that it can be incorporated into the treatment programs of these individuals.

## 1. Introduction

Obesity, a disorder involving excessive body fatness, affects approximately 650 million individuals around the world [1]. The pathophysiology of obesity is complex and involves several body systems [2]; indeed the altered physiological processes occurring in this disorder may lead to long-term complications such as cardiovascular and metabolic diseases, osteoarthritis, and gastrointestinal disorders, as well as an increased risk of death [2]. Regular physical exercise reduces the complications of obesity, resulting in a better quality of life and a reduced risk of concomitant diseases and death [3]. However, individuals with obesity have low adherence to regular physical exercise [4]. Therefore, it is necessary to assess the clinical relevance and acceptance of different exercise modalities, to optimize the treatment protocols for populations with physical limitations. The combination of different exercise modalities can improve obesity-related outcomes, as it is possible to observe in the current review.

Vibratory therapy is an intervention, in which mechanical vibration produced in a device is transmitted to a part or the whole body of the individual. When mechanical vibration is generated by a device and applied to a specific region of the body, the intervention is referred to as local vibratory therapy [5]. When the individual is exposed to mechanical vibration generated in a vibrating platform (VP) and it is transmitted to the whole body of the individual, whole-body vibration (WBV) is produced, and this intervention would be considered systemic vibratory therapy [6,7,8].

WBV seems to stimulate reflexive muscle contractions in a gentle manner [9,10]. Moreover, WBV has been demonstrated to be a safe and effective intervention for individuals with obesity, contributing to reductions in fat mass [11], improvements in functional capacity [12,13], enhancements in muscular strength and flexibility [14], better cardiovascular function [15], and increased sleep quality [16]. WBV has been applied as an adjunct to various therapeutic interventions in different populations, demonstrating the potential to optimize clinical outcomes [17,18,19,20,21]. Although less studied, the combination of WBV with other exercise modalities in overweight and obese subjects has also been evaluated. For instance, WBV combined with high-intensity interval training (HIIT) was shown to enhance the reductions in fat mass, triglycerides, and total cholesterol rates in obese adults [22]. Additionally, the combination of WBV with maximal voluntary contractions improved functional parameters in obese adolescent males [23]. Wilms et al. (2012) [24] studied the concurrent performance of endurance training and WBV in obese women, and increased resting energy expenditure and an improvement in the phase angle, assessed by bioelectrical impedance analysis, were observed after the combined intervention. Moreover, Figueroa et al. (2012) [25] found improvements in cardiovascular function and muscle strength after 6 weeks of WBV combined with an external load in overweight and obese women. Despite these positive outcomes, this review highlights considerable variability in biomechanical parameters, vibration exposure time, number of bouts, participant positioning, and the diversity of outcomes assessed. The methodological inconsistency of these studies does not yet allow us to define which are the most appropriate biomechanical parameters for the clinical application of WBV in overweight and obese individuals. Considering the heterogeneity of the studies, this systematic review aimed to summarize the WBV protocols employed and the physiological effects of WBV combined with other exercise modalities in overweight and obese individuals.

## 2. Methods

The current systematic review followed the Preferred Reporting Items for Systematic Reviews and Meta-Analysis (PRISMA) guidelines [26]. This is a qualitative systematic review, and no meta-analysis was conducted due to heterogeneity in study designs and outcome measures. This study was registered in the International Prospective Register of Systematic Reviews (PROSPERO) with the number CRD42020210109 [27].

### 2.1. Search Strategy

A comprehensive literature search was conducted in the electronic databases PubMed, Embase, Web of Science, Scopus, and Cochrane up to 21 March 2023. The following keywords were utilized: (“whole body vibration” OR “vibration exercise” OR “vibration training”) AND (“combined” OR “combination” OR “combining” OR “added” OR “associated” OR “association”) AND (“overweight”) AND (“obesity” OR “obese”). The keywords used were defined based on the following question: “What are the effects of combining WBV with other modalities of physical exercise in individuals with overweight and obesity?”; they were defined according to the PICO strategy, focusing on overweight and obese patients (P-Participants) and their relationship to the combination of WBV with other physical exercise modalities (I-Intervention) without restrictions regarding comparisons (C-Comparison). All reported outcomes (O-Outcomes) were considered relevant to the studied population [28].

### 2.2. Eligibility Criteria

Randomized controlled trials (RCTs) that investigated effects of the combination of WBV with other physical exercise modalities in overweight (body mass index (BMI) 25–30 kg/m^2^) and obese (BMI > 30 kg/m^2^) individuals were evaluated. All studies that combined WBV with other physical exercise (e.g., squat exercise, resistance exercise, maximal voluntary contraction, or high-intensity interval training) independent of the type, duration, or intensity of exercise protocols applied, or the year of the publication, were included in this systematic review.

Studies were excluded if they (i) were editorials, letters, conference abstracts, reviews, commentaries, or short communications; (ii) were published in a language other than English; (iii) had findings not related to obesity or being overweight and WBV; and (iv) had WBV as an intervention, but it was not combined with other modalities of exercises.

**Figure 1:**
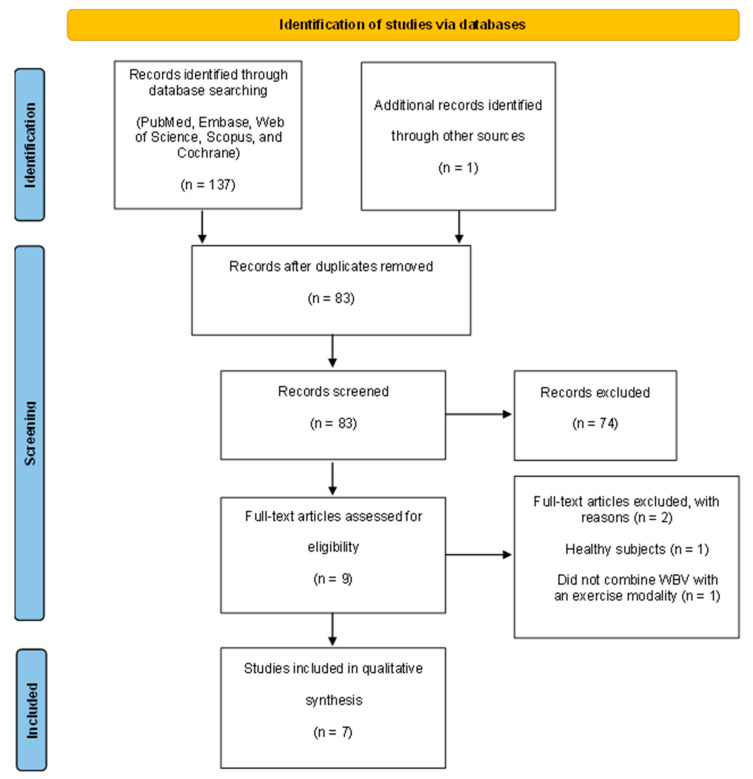
PRISMA flow diagram of the literature selection process.

A flowchart (Figure 1), developed in accordance with the PRISMA guidelines, illustrates the selection process of the full-text articles included in the current systematic review [26].

### 2.3. Methodological Quality

The PEDro scale was applied to verify the methodological quality of the included studies (http://www.pedro.org.au/english/downloads/pedro-scale/ [accessed on 20 September 2023]), which consists of eleven items. The selected articles with a score of seven or greater on the PEDro scale were considered of ‘high’ methodological quality, those with a score of five to six of ‘fair’ quality, and with a score of four or below of ‘poor’ quality [29,30].

### 2.4. Risk of Bias

The Cochrane Collaboration’s tool was utilized to assess the risk of bias in the included articles [31]. For all the assessments, each manuscript was assigned to one reviewer (DBS), cross-checked by a second reviewer (ACCO), and in case of disagreement, a third reviewer was consulted (DCS-C), and the issue was discussed until consensus was reached (AW, DCSC, and MB-F).

### 2.5. Study Selection and Data Extraction

The systematic review followed four steps: identification, screening, eligibility, and data extraction. In the identification step, records were identified through a database search, and duplicates were removed. Two reviewers (DB and ACO) independently screened titles and abstracts, and excluded irrelevant studies based on eligibility criteria after reading the references. For eligibility, relevant full texts were analyzed, and all applicable works were included in the systematic review. A third reviewer resolved any disagreements (VA). The researchers conducted the data extraction phase, where they collected information from the included publications. They extracted details such as author and year, demographic data, study design, objectives, WBV protocols, intervention combinations, and outcomes.

## 3. Results

### 3.1. Literature Selection Process

A total of 137 studies were identified through the database search, with an additional study sourced from other references. Following the removal of 55 duplicate records, 83 studies remained for the screening phase. During the screening process, 74 publications were excluded as they did not pertain to the research question. Subsequently, the full texts of nine studies were reviewed in detail. After a careful analysis, two studies were excluded for not evaluating the effect of WBV in combination with another modality of exercise. In conclusion, seven studies were incorporated in this systematic review. Figure 1 summarizes the selection process.

### 3.2. Methodological Quality

**Figure 2:**
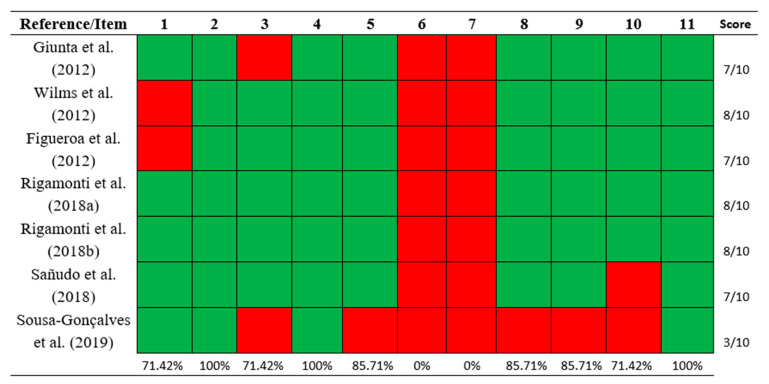
Methodological quality assessment of included studies with the PEDro scale [22,23,24,25,32,33,34]. (1) Eligibility criteria were specified; (2) subjects were randomly allocated to groups; (3) allocation was concealed; (4) groups were similar at baseline regarding the most important prognostic indicators; (5) there was blinding of all subjects; (6) there was blinding of all therapists who administered the therapy; (7) there was blinding of all assessors who measured at least one key outcome; (8) measures of at least one key outcome were obtained from more than 85% of the subjects initially allocated to groups; (9) all subjects for whom outcome measures were available received treatment or control condition as allocated or, where this was not the case, data for at least one key outcome was analyzed by “intention to treat”; (10) results of between-group statistical comparisons are reported for at least one key outcome; and (11) study provides both point measures and measures of variability for at least one key outcome. Green color = criterion was clearly satisfied. Red color = criterion was not clearly satisfied.

The included publications demonstrated a mean methodological quality score of 7, as assessed by the PEDro scale (Figure 2), with scores ranging from a minimum of 3 to a maximum of 8, indicating an overall moderate methodological quality. Six studies [22,24,25,32,33,34] were considered “high” quality, and one study [23] was “poor” quality.

### 3.3. Main Findings and Intervention Protocols

Table 1 summarizes the participant characteristics, study objectives, and outcomes of the included articles. Table 1 demonstrates that (i) WBV stimulates GH release (*p* < 0.05) [32,33,34] and lactate production (*p* < 0.05) [34]; (ii) WBV combined with endurance training increases the bioelectrical phase angle (*p* = 0.04) [24]; (iii) WBV added to HIIT reduces (*p* < 0.05) fat mass, blood triglycerides, and cholesterol concentrations [22]; (iv) acutely, the combination of WBV with MVC does not significantly affect systolic blood pressure (SBP), diastolic blood pressure (DBP), mean arterial pressure (MAP), and peripheral oxygen saturation (SpO2) [23]. However, longitudinal research shows that WBV combined with resistance exercise effectively reduces (*p* < 0.05) SBP, arterial stiffness, wave reflection magnitude, and heart rate, and balances the cardiac autonomic function [25]. The number of participants in the included studies ranged from 7 up to 40 individuals, with a mean age of 24.8 years old (ranging from 17.1 to 43.1 years old). Several outcomes were assessed: (i) GH release [32,33,34], lactate production [34], blood lipids (total cholesterol, high-density lipoprotein cholesterol, low-density lipoprotein, and triglycerides), and plasma glucose [22] via blood sampling; (ii) bioelectrical phase angle (PhA), body composition, and resting energy expenditure using a bioelectrical impedance analysis (BIA) [22,23,24]; and (iii) blood pressure monitored by a sphygmomanometer, body mass, and height measured using a portable stadiometer [23].

Table 2 summarizes the WBV intervention protocols utilized across the included studies. The type of VP most used in the studies was the platform with vertical vibration [23,24,32,33,34]; one study used the alternating platform [22], and one study did not specify the method [25]. The frequencies used in the studies varied from 18 to 35 Hz and the peak-to-peak displacement varied from 2 to 5 mm. The studies combined SVT with static and dynamic squatting [25,34], endurance training [24], maximal voluntary contraction [23,32,33], and high-intensity interval training [22]. When comparing studies with similar outcomes, a frequency range of 30–35 Hz appears to be associated with an acute increase in GH release.

In accordance with the reporting guidelines for WBV studies [6], the present review found that the included studies reported several key items recommended in the checklist for human WBV research. These are the types of vibrating platforms, biomechanical parameters of the mechanical vibration, the positioning of the individual on the vibrating platform, and the duration of the sessions. However, several other critical items are frequently absent from many of the included studies, such as the environmental conditions, a detailed description of the vibrating platform, whether the biomechanical parameters were verified through accelerometry, whether the individuals were wearing footwear, and if and how skidding of feet was prevented. Moreover, if the transmission of mechanical vibration to the head of the individuals was prevented, if a supervisor was present during the sessions, and if the individuals had previous experience or side effects with WBV were not included. These items may directly influence the results and reproducibility of the research, as well as the safety of individuals, and should be reported in future studies.

### 3.4. Risk of Bias

**Figure 3:**
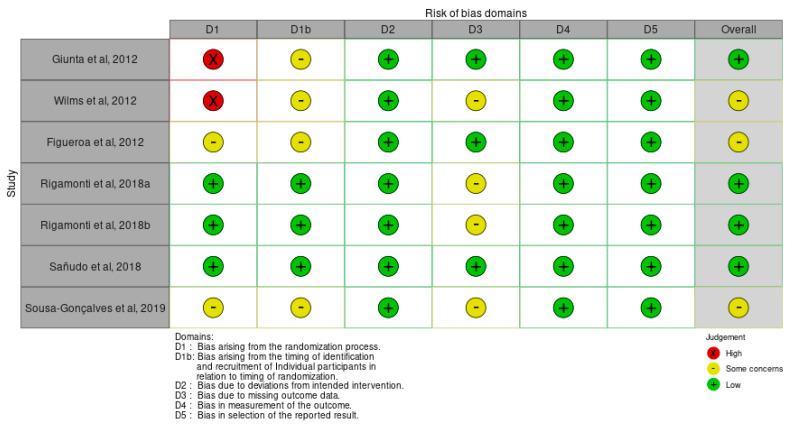
Risk of bias of selected publications [22,23,24,25,32,33,34].

The risk of bias of the included studies was assessed with the Cochrane risk of bias tool (Figure 3). Four studies were at a low risk of bias and three studies were at a moderate risk of bias. The main sources of bias found were insufficient descriptions of the randomization process, identification, and recruitment of participants in relation to randomization, and missing outcome data.

## 4. Discussion

The current systematic review aimed to investigate the effects of the combination of WBV with other modalities of exercises in overweight and obese individuals. The methodological quality of the studies included was moderate, mainly regarding the random sequence generation, concealed allocation, lack of blinded subjects, therapy administrators, and referred assessors in outcome measurements.

The results showed that the concurrent use of WBV with other modalities of exercises may lead to physiological responses related to GH (post-exercise), body composition, and blood lipid levels, as well as to cardiovascular function in overweight and obese individuals.

This review found that, of the seven studies included, four studies addressed acute effects, and three studies investigated cumulative effects. Therefore, it was not possible to verify whether the acute effects found were maintained over time and whether the continuity of the stimuli potentiates the results. In addition, there is variability in the WBV protocols used (types of vibrating platforms, time of exposure to vibration, number of bouts, and positioning of the individual), as well as the diversity of the outcomes analyzed. In this context, it is impossible to define which protocol would be most appropriate to achieve the best results for each clinical outcome studied. Furthermore, the moderate methodological quality and risks of bias found in the included studies partly affect the reliability of the results obtained.

### 4.1. Effects on GH Concentrations

Three publications reported the effects of a combination of WBV with other modalities of exercises on GH concentration in obese individuals. WBV combined with squats plus an external load did not produce an additive acute effect on GH concentrations (post-exercise) compared to WBV alone [34]. In contrast, the addition of WBV to MVC resulted in an immediate increase in GH levels post-intervention [32,33]. Both studies showed the positive effects of WBV using 2.85 g of acceleration and frequencies of 30 Hz [34] and 35 Hz [32,33]; however, the results suggest that the combination of WBV with MVC is more effective.

Previous studies have demonstrated the effects of WBV on GH levels in diverse populations [35]. Sartorio et al. (2011) [36] investigated the effects of WBV combined with MVC in healthy adults and found a greater effect of this combination on GH release compared to WBV alone. Kvorning et al. (2006) [37] investigated the combination of WBV with resistance training in healthy young men and observed higher GH release levels when those exercises were combined. Paineiras-Domingos et al. (2017) [35], in a systematic review, reported that WBV increased GH concentrations in all reviewed studies except for two papers published by Cardinale et al. (2010) [38] and Di Loreto et al. (2004) [39]. Two factors that may be related to the results of these two studies are the frequency utilized and the age of the individuals. These two studies utilized the frequency of 30 Hz and were conducted with older populations compared to the other studies. Paineiras-Domingos et al. (2017) [35] concluded that due to the variety of frequencies used in the studies, the GH response does not seem to depend directly on this parameter.

The mechanism related to the neuroendocrine responses attributed to WBV is not fully understood. It is viable that vibration stimulates peripheral mechanoreceptors and promotes muscle activation that sends afferent signals toward the central nervous system and promotes the response of the hypothalamic–pituitary–adrenal axis, favoring metabolic regulation.

In overweight and obese individuals, the GH has potent anabolic, lipolytic, and anti-inflammatory actions [33,40] that act on fat metabolism, prevent endothelial dysfunction and premature atherosclerosis, and improve cardiovascular modulation and systolic function of the heart. These factors are important for reducing cardiovascular risk factors in this population [40].

Given the current paucity of evidence, additional well-designed studies are warranted to elucidate which combinations of exercise modalities and protocol parameters are most effective in enhancing GH concentrations among overweight and obese individuals.

### 4.2. Effects on Body Composition

Two studies examined the effects of combining WBV with other exercise modalities on body composition in obese individuals. WBV combined with HIIT and a hypocaloric diet for 8 weeks in obese adults reduces body fat compared to the group without vibration [22]. WBV combined with an endurance training program in obese women increased the PhA compared to endurance training alone [24]. The PhA is an important parameter that represents an indicator of cellular health in chronic inflammatory states [41]. Vertical [24] and side-alternating [22] platforms were used in these investigations and the duration of the protocols was 6–8 weeks [22,24]. Despite the positive results, it is suggested that protocols with a longer duration would contribute to improvements in body composition. Moreover, acute studies on this population may bring additional information.

The benefits of WBV alone in terms of body composition have been described [24,42,43,44,45,46,47]. Vissers et al. (2010) [43] observed that WBV was more effective than aerobic exercise in reducing visceral adipose tissue in obese individuals. Obese women that performed WBV had significantly lower BMIs, total body and trunk fat, sum of skinfolds, and body circumferences [46]. Moreover, a 12-week WBV intervention on body composition in individuals with type 2 diabetes caused a decrease in body mass, waist circumference, waist-to-hip ratio, and body fat [45]. An increase in bone mineral density (BMD) was observed when combining WBV and resistance training for 8 months in obese postmenopausal women [47] and after 18 weeks of WBV combined with cyclic hypoxia in elderly participants [19].

One possible mechanism associated with improvements in body composition after the combination of WBV with other exercise modalities may be an increase in GH release. Studies show that adequate GH levels are associated with reduced cardiovascular risk factors in obese individuals, such as body mass, visceral fat and fat mass, and lipid profile [48,49]. High insulin and low GH levels are frequently observed in obesity, with reduced energy expenditure and further fat accumulation [50]. In addition, studies demonstrated that WBV combined with cyclic hypoxia [19], lower limb strengthening [51], auriculotherapy [52], and parathyroid hormone [20] promote an increase in functionality in different populations. This enhancement in functionality probably contributes to the improvements in body composition, as the individuals become more active and may increase their daily caloric expenditure.

### 4.3. Effects on Blood Lipids and Cardiovascular Markers

Sañudo et al. (2018) [22] enrolled obese adults into a protocol of WBV added to HIIT and a hypocaloric diet. Reductions in blood triglycerides and cholesterol concentrations were observed compared to HIIT and diet or diet alone. Miyaki et al. (2012) [44] combined WBV with lifestyle modifications in overweight and obese women, and the association reduced triglycerides and LDL cholesterol, but the effects of WBV alone were not measured. Bellia et al. (2014) [53] combined WBV with dietary interventions in middle-aged obese subjects and found improvements in fasting insulin, leptin, and adiponectin concentrations. These findings suggest that combining WBV with other exercises and dietary re-education may enhance the reduction in lipid levels and other blood markers related to obesity.

Regarding cardiovascular outcomes, there were no significant variations in SBP, DBP, MAP, and SpO2 after an acute intervention combining WBV with MVC compared to MVC alone; only an increase in heart rate was found [23]. However, a 6-week protocol of WBV combined with dynamic and static semi-squats, wide-stand semi-squats, and calf-raises reduced the wave reflection magnitude, arterial stiffness, and SBP [25]. Moreover, there was a significant decrease in the resting heart rate, which resulted from a decline in sympathovagal balance (a concurrent drop in sympathetic modulation and an increase in cardiovagal modulation) [25]. These findings suggest that WBV is a low cardiovascular stress intervention for overweight and obese individuals, as it does not induce significant acute changes in heart-related markers and may provide long-term cardiovascular benefits.

The search for safe exercises for individuals with cardiovascular risk factors is an important aim for clinical researchers. WBV has been investigated for this purpose and represents a promising alternative for obese individuals and other populations [19,54]. Severino et al. (2017) [15] observed improvements in heart rate variability in obese Hispanic postmenopausal women and Licurci et al. (2018) found similar effects in elderly subjects [54] submitted to WBV. Wong et al. (2016) [18] found improvements in nitric oxide levels and BP after 8 weeks of WBV in obese postmenopausal women. These findings suggest that, in the long-term, WBV improves cardiovascular markers for populations with an elevated cardiovascular risk. To date, only the study by Figueroa et al. (2012) [25] evaluated the effectiveness of WBV as an adjunct to other exercise types for the improvement of cardiovascular markers and showed promising results. Consequently, further research on this topic is warranted.

### 4.4. Strengths

The strengths of the current review are the methodological rigor with adherence to recognized guidelines; topic with clinical and social relevance; potentially valuable contributions to public health; identification of gaps in the literature and directions for future research; and summary of the effects of WBV combined with other exercise modalities in overweight and obese individuals.

### 4.5. Limitations

The limitations of the present work include the following: (i) the small sample sizes of included studies; (ii) the evaluation of only serum GH levels while disregarding other hormones known to be affected by WBV such as cortisol [38]; (iii) most studies included in this systematic review were acute; (iv) the heterogeneity of the protocols of the selected publications; (v) the insufficient reporting of WBV protocols in the included studies; and (vi) only papers in the English language were included.

## 5. Conclusions

The combination of WBV with other modalities of exercise may be a promising intervention to promote physiological responses to GH, body composition, and blood lipid levels, as well as to cardiovascular function in overweight and obese individuals. Based on these findings, it is suggested that WBV be included in existing treatment programs for this population. However, considering the small sample sizes, the predominance of acute studies, and the moderate methodological quality of the included research, more large-scale, long-term, randomized controlled trials with interventions with WBV and other modalities of exercise on physiological parameters in overweight and obese cohorts are needed to corroborate and expand the findings of the current systematic review.

Clinical relevance: WBV seems to stimulate neuroendocrine responses, including GH release, and may contribute to favorable modifications in body composition parameters, such as the bioelectrical phase angle, total and trunk fat mass, sum of skinfolds, and body circumferences. Additionally, WBV has been associated with improvements in cardiovascular markers among populations at an elevated cardiovascular risk. WBV represents a potentially promising adjunctive strategy for the management of overweight and obese individuals.

## Figures and Tables

**Table 1:** Main findings of selected studies.

Study	Study Design	Demographics	Aim	Primary Outcomes	Interventions	Results
Giunta et al. (2012) [34]	A randomized cross-over trial	n = 7, severely obese women, age = 22 ± 5 years old, BMI: 39.9 ± 2.9 kg/m^2^, two groups = WBV and WBV + S	To evaluate acute effects of WBV on GH responses in severely obese females.	Serum GH levels and blood lactate concentrations.	WBV combined with squatting plus external load	WBV independently stimulates GH secretion and lactate production; however, no additional effects are observed when it is combined with squatting performed with external load.
Wilms et al. (2012) [24]	A randomized controlled trial	n = 14, obese women, age = 43.1 ± 3.5 years old, BMI: 37.4 ± 1.3 kg/m^2^, two groups = WBV and non-WBV	To explore effects of WBV added to an endurance training program on the bioelectrical phase. angle and body composition parameters as well as on REE in obese women that were not set on a calorie-restrictive diet.	Bioelectrical phase angle, body composition and resting energy expenditure.	WBV combined with endurance training	WBV combined with endurance training increased the bioelectrical phase angle in obese women.
Figueroa et al. (2012) [25]	A randomized cross-over trial	n = 10, young obese/overweight and normotensive women, age = 22.4 ± 1.8 years old, BMI: 29.9 ± 0.8 kg/m^2^, two groups = WBV and CON	To evaluate the effectiveness of a 6-week WBV training program on arterial function, autonomic function, and muscle strength in young overweight/obese women.	Arterial stiffness, blood pressure and sympathovagal balance.	WBV combined with dynamic and static semi-squats, wide-stand semi-squats, and calf-raises with external load	WBV decreased systemic arterial stiffness and SBP via improvements in wave reflection and sympathovagal balance in young overweight/obese normotensive women.
Rigamonti et al. (2018a) [32]	A randomized cross-over trial	n = 8, obese male adolescents, age = 17.1 ± 3.3 years old, BMI: 36.5 ± 6.6 kg/m^2^, three groups = WBV, MVC, and WBV + MVC	To evaluate the GH response to MVC combined with WBV (MVC + WBV), compared to those after MVC or WBV alone, in a group of obese adolescents. Changes in serum cortisol and IGF-I and blood lactate levels were also evaluated.	Serum GH isoforms levels.	WBV combined with MVC	GH peaks and nAUCs after MVC + WBV and MVC were significantly higher than WBV alone, without any difference between MVC + WBV and MVC groups. Anyway, GH levels immediately after execution of the exercise were significantly higher when obese subjects were administered with MVC + WBV than MVC alone.
Rigamonti et al. (2018b) [33]	A randomized cross-over trial	n = 8, obese male adolescents, age = 17.1 ± 3.3 years old, BMI: 36.5 ± 6.6 kg/m^2^, three groups = WBV, MVC, and WBV + MVC	To measure circulating levels of 22 kDa-GH and 20 kDa-GH in a cohort of obese subjects undergoing different protocols of exercise at increasing intensity WBV and MVC.	Serum GH, cortisol, IGF-I, and blood lactate levels.	WBV combined with MVC	The concomitant application of WBV and MVC elicits the greatest responses in both GH isoforms.
Sañudo et al. (2018) [22]	A randomized controlled trial	n = 40, obese/overweight adults, three groups = HIITWBV (age = 35 ± 7 years old, BMI: 31.2 ± 4.0 kg/m^2)^, HIIT (age = 35 ± 7 years old, BMI: 32.3 ± 5.4 kg/m^2^), CG (age = 36 ± 9 years old, BMI: 31.2 ± 5.0 kg/m^2^)	To compare the effect of HIIT with additional vibration recovery on body composition and health-related parameters in obese/overweight adults who were placed on a hypocaloric diet.	Body composition and biochemical indices.	HIIT combined with WBV and diet restriction	The addition of WBV to HIIT combined with a hypocaloric diet leads to greater reductions in fat mass, blood triglyceride levels, and cholesterol concentrations compared to HIIT with diet or diet alone.
Sousa-Gonçalves et al. (2019) [23]	A randomized cross-over trial	n = 8, obese male adolescents, age = 17.1 ± 3.3 years old, BMI: 36.5 ± 6.6 kg/m^2^, three groups = WBV, MVC, and MVC + WBV	To evaluate the acute effects of WBV and MVC, alone and in combination, on some parameters of cardiorespiratory and MSMF in obese adolescents.	Cardiorespiratory, musculoskeletal and neuromotor fitness.	WBV combined with MVC	No significant changes were observed in SBP, DBP, MAP, and SpO2 after the 3 tests, and only a significant HR increase was observed after MVC + WBV and MVC alone.

BMI—body mass index, WBV—whole-body vibration, S—squat, GH—growing hormone, REE—resting energy expenditure, MVC—maximal voluntary contraction, CON—control, nAUCs—net areas under the curve, MSMF—musculoskeletal and neuromotor fitness, SpO2—peripheral oxygen saturation, HIIT—high-intensity interval training, SBP—systolic blood pressure, DBP—diastolic blood pressure, MAP—mean arterial pressure.

**Table 2:** Intervention protocols used in included studies.

Study	WBV Intervention	Parameters	Type of Vibrating Platform	Positioning	Steps of the Protocol
Giunta et al. (2012) [34]	Acute effect. (i) WBV group; (ii) WBV + squat group	Frequency 30 Hz, acceleration 2.85 g	Vertical vibration	WBV group—static squat/WBV + squat group—dynamic squats with an additional external load (contained in a vest, range 18–23 kg) corresponding to 40% of the FFM	WBV group—10 bouts of 72 s with 50-s rest in between/WBV + squat—10 series of 12 dynamic squats with 50 s rest in between. Total—19 min and 30 s.
Wilms et al. (2012) [24]	6 week intervention. (i) endurance training group and (ii) endurance training + WBV group	Peak-to-peak displacement 2 mm, frequency 30 Hz.	Vertical vibration	Static exercises for different muscle groups: (i) the first week: squats, lunges, biceps curls, and shoulder relaxation; (ii) second week: complemented by exercises for the sural muscle and one-leg stands; (iii) third week: complemented by exercises for the abdominal side muscles, triceps curls, and side crunches; and (iv) fourth week: complemented by press-ups, and exercises for the lower abdominal muscles and pelvis muscles.	Work time was 30 s with 30 s rest. 5 min in week 1, 8 min in week 2, 13 min in week 3, and 16 min from week 4 onwards.
Figueroa et al. (2012) [25]	6 weeks intervention. (i) WBV + external load group and (ii) control group	Frequency 25–30 Hz, peak-to-peak displacement 2–4 mm, acceleration 2.83–4.86 g	Not specified	Dynamic and static semi-squats with a 120° knee angle (considering 180° as full knee extension), wide-stand semi-squat, and calf-raises.	The dynamic exercises were performed with slow movements at a rate of 2 and 3 s for concentric and eccentric phases, respectively. The vibration intensity progressed by increasing the frequency (25–30 Hz) and amplitude (1–2 mm). The duration of the sets and rest periods was progressively increased (30–60 s) and decreased (60–30 s), respectively. During the last 2 weeks, subjects used a weight vest with 5% and 10% of their body weight during weeks 5 and 6, respectively.
Rigamonti et al. (2018a) [32]	Acute effect. (i) WBV group; (ii) MVC group; and (iii) MVC + WBV group	Frequency 35 Hz, peak-to-peak displacement 5 mm, acceleration 2.85 g	Vertical vibration	The subject stood on a vibrating platform with the knees at 110°.	15 bouts of the 30 s of work time with 30 s rest. A total of 15 min.
Rigamonti et al. (2018b) [33]	Acute effect. (i) WBV group; (ii) MVC group; and (iii) MVC + WBV group	Frequency 35 Hz, peak-to-peak displacement 5 mm, acceleration 2.85 g	Vertical vibration	The subject stood on a vibrating platform with the knees at 110°.	15 bouts of the 30 s of work time with 30 s rest. A total of 15 min.
Sañudo et al. (2018) [22]	8 weeks intervention. (i) HIIT group; (ii) HIIT + WBV group; and (iii) Control group	Frequency 18–25 Hz, peak-to-peak displacement 4 mm, acceleration 2.6 g	Side-alternating platform	Isometric squat position, approximately 100° knee flexion lightly holding the handrails, wearing sport footwear	3 times per week for 8 weeks (with at least 1 rest day between sessions) and performed 6 sets × 1 min of HIIT at 90% HRpeak followed by 6 × 1 min of interset vibration. Training volume increased by 1 set every 2 weeks until 10 sets at week 8 and vibration was increased similarly to HIIT bouts until 25 Hz (4 mm)
Sousa-Gonçalves et al. (2019) [23]	Acute effect. (i) WBV; (ii) MVC; and (iii) MVC + WBV	Frequency 35 Hz, peak-to-peak displacement 5 mm, acceleration 2.85 g	Vertical vibration	Static squat position with 110° knee flexion	15 bouts of 30 s without rest

WBV—whole-body vibration, g—multiple of gravity, MVC—maximal voluntary contraction, FFM—fat-free mass, HIIT—high-intensity interval training, HR—heart rate.

## Data Availability

No new data were created.
